# Male-killer symbiont screening reveals novel associations in *Adalia* ladybirds

**DOI:** 10.1099/acmi.0.000585.v3

**Published:** 2023-07-07

**Authors:** Jack Archer, Gregory D. D. Hurst, Emily A. Hornett

**Affiliations:** ^1^​ Institute of Infection, Veterinary and Ecological Sciences, University of Liverpool, Liverpool, UK; ^2^​ Vector Biology, Liverpool School of Tropical Medicine, Liverpool, UK

**Keywords:** endosymbiont, *Spiroplasma*, ladybird, *Adalia*, male-killing, cobiont

## Abstract

While male-killing bacteria are known to infect across arthropods, ladybird beetles represent a hotspot for these symbioses. In some host species, there are multiple different symbionts that vary in presence and frequency between populations. To further our understanding of spatial and frequency variation, we tested for the presence of three male-killing bacteria: *Wolbachia *, *Rickettsia* and *Spiroplasma *, in two *Adalia* ladybird species from a previously unexplored UK population. The two-spot ladybird, *A. bipunctata*, is known to harbour all three male-killers, and we identified *Spiroplasma* infection in the Merseyside population for the first time. However, in contrast to previous studies on two-spot ladybirds from continental Europe, evidence from egg-hatch rates indicates the *Spiroplasma* strain present in the Merseyside population does not cause embryonic male-killing. In the related ten-spot ladybird, *A. decempunctata*, there is only one previous record of a male-killing symbiont, a *Rickettsia *, which we did not detect in the Merseyside sample. However, PCR assays indicated the presence of a *Spiroplasma* in a single *A. decempunctata* specimen. Marker sequence indicated that this *Spiroplasma* was divergent from that found in sympatric *A. bipunctata*. Genome sequencing of the *Spiroplasma *-infected *A. decempunctata* additionally revealed the presence of cobionts in the form of a *Centistes* parasitoid wasp and the parasitic fungi *Beauveria*. Further study of *A. decempunctata* from this population is needed to resolve whether it is the ladybird or wasp cobiont that harbours *Spiroplasma *, and to establish the phenotype of this strain. These data indicate first that microbial symbiont phenotype should not be assumed from past studies conducted in different locations, and second that cobiont presence may confound screening studies aimed to detect the frequency of a symbiont in field collected material from a focal host species.

## Data Summary

The authors confirm all supporting data, code and protocols have been provided within the article or through supplementary data files. Supporting data has been deposited to Figshare with the following DOIs: PCR primer sequences and conditions (https://doi.org/10.6084/m9.figshare.21915558.v1 [1]); individual sample collection and screening data (https://doi.org/10.6084/m9.figshare.21865035 [2]); *Spiroplasma* genome annotations (https://doi.org/10.6084/m9.figshare.21865113.v1 [3]). Four *Spiroplasma* 16S rRNA sequences have been deposited in GenBank (accessions: OQ271402-OQ271404; OQ271406). Further sequencing data has been deposited within BioProject PRJNA921942 to GenBank: whole-genome sequencing data for one *Adalia decempunctata* specimen (SRR23019831), and draft genome assemblies for *Spiroplasma* sp. (SAMN32746590) and *Beauveria* sp. (SAMN32746591).

## Introduction

Arthropods are commonly infected with bacterial endosymbionts that have a profound impact upon their biology; affecting host physiology, development and susceptibility to natural enemies [4]. These effects on individuals can ultimately influence the dynamics of their host population and communities [5], reproductive behaviour [6, 7], and host evolution [8, 9]. The influence of maternally inherited endosymbionts is particularly pronounced as their mode of transmission produces a selective pressure on the symbiont to manipulate host reproduction to favour females. Several reproductive manipulations have been recorded, including cytoplasmic incompatibility (CI), parthenogenesis induction (PI), feminization of genetic males, and male-killing (MK) [8, 10].

Male-killing endosymbionts – microbes that kill male host offspring, usually early in development during embryogenesis – are particularly important for two main reasons. First, when they become common, they distort the population sex ratio and this can influence host reproductive ecology. In butterflies, for instance, high prevalence of male-killers in the host population is variously associated with increased female remating rate [7], reduced female capacity to encounter mates [11], and sex role reversal [6]. In ladybirds, the population sex ratio bias associated with a high frequency of MK microbes is sufficient to alter the dynamics of sexually transmitted infections in the host [12]. Where the MK microbe is common, females greatly exceed males in the population, and the mean mating rate of male ladybirds therefore exceeds that of female. This disparity of mating rate is reflected in epidemics of the sexually transmitted mite *Coccipolipus hippodamiae* in which male ladybirds become infected before females [12]. Second, male-killers are parasites, and may engender strong selective pressure upon their host to suppress their action. Indeed, the intense Fisherian selection for restoration of the rare sex make selection to suppress male-killer activity amongst the strongest observed in natural systems [13, 14]. It is also hypothesized that selection for suppression may act on the sex determination system of the host, and thus represent a driver of sex determination system evolution [9].

While MK microbes are known to infect a range of arthropods, members of the family Coccinellidae (ladybird beetles) are particularly commonly infected, albeit with infection usually being at low-mid prevalence [15]. Male-killers in ladybirds derive from diverse microbial groups, including bacteria from the flavobacteria, Mollicutes, ɣ-proteobacteria and α-proteobacteria [16]. Breeding data alongside molecular genetic analysis has demonstrated that the two-spot ladybird *Adalia bipunctata* is infected with three heritable MK bacteria: *Spiroplasma *, *Rickettsia* and *Wolbachia* [17–19]. These vary in presence and frequency between populations. For instance, the *Rickettsia* male-killer has been found broadly in the UK, Germany, The Netherlands, Denmark and Russia, but is commonly at low frequency (<20 % of females infected [17, 19, 20]). *Spiroplasma *, by contrast, varies greatly in frequency, being rare in Germany (<20 % of females infected) but common in St. Petersburg, Russia (>20 % of females infected), and Stockholm, Sweden (>50 % of females infected [12, 19, 20]). *Wolbachia* MK infections have been reported solely in the Eastern part of the range, in Moscow [18, 20], although *Wolbachia* of unknown phenotype have also been found in two-spot ladybirds from Stockholm [12]. The causes of variation in the presence and prevalence of different male-killers across *A. bipunctata* populations is poorly understood.

In contrast, there is only one report of a MK infection in the congeneric and often sympatric ten-spot ladybird, *Adalia decempunctata*: a *Rickettsia *. Present in 4–6 % of female beetles in two German populations, infected females produced strongly female biassed broods that also carried the *Rickettsia* through vertical transmission [21]. This previous work also noted the absence of *Spiroplasma* and *Wolbachia* in these *A. decempunctata* populations. While *Rickettsia* was also noted in ten-spot ladybirds from Stockholm (4 of 18 females were infected), it is unknown whether this strain also causes MK [22]. Surveys for MK symbionts have been conducted less broadly in *A. decempunctata* than in *A. bipunctata,* making it unclear whether differences between the species reflect intensity of study, breadth of populations studied, or real difference in the diversity and frequency of symbiont infection.

To further investigate the incidence of MK in *Adalia* ladybirds we tested for the presence of *Wolbachia *, *Rickettsia* and *Spiroplasma* in *A. bipunctata* and *A. decempunctata* from a previously un-investigated population in the UK (the Merseyside region). This represents a third population study for the ten-spot ladybird, and a further geographical sample for the two-spot ladybird to that previously studied. We identified *Spiroplasma* in this population of *A. bipunctata* and examined whether infection is associated with embryonic MK activity, as observed elsewhere. We also report an *A. decempunctata* individual that tested positive for *Spiroplasma *, with onward genomic analysis of this association.

## Methods

### Sample collection

Thirty-seven adult *A. bipunctata* (15 male, 22 females) and 40 adult *A. decempunctata* (20 males, 20 females) were collected by eye from Liverpool in March 2022 (*A. decempunctata* and *A. bipunctata*), and from Liverpool and the Wirral in May–June 2022 (*A. bipunctata* only). Material collected in March derived from overwintering sites (gravestones in Anfield Cemetery). Material collected in May–June derived from lime trees (*Tilia europea*), rose (*Rosa sp*.) and nettle (*Urtica dioica*). Adults were sexed morphologically following characteristics in Randall *et al.* [23]. Details of beetle collection times and locations can be found in supporting data (https://doi.org/10.6084/m9.figshare.21865035 [2]).

### Embryonic male-killing phenotype in *A. bipunctata*

Twenty female *A. bipunctata* from Liverpool and Wirral were individually placed at room temperature in petri dishes along with filter paper on which to lay eggs, supplied with aphids to eat, and a male *A. bipunctata* to maintain fertility. Each day, any egg clutches laid were removed, the eggs counted, and placed in empty labelled petri dishes. At 5–6 days post-oviposition, the number of hatched eggs were scored. From these data, the proportion of eggs that hatched per clutch was calculated (hatched eggs/total eggs). These data were combined with later infection data of the mothers gained from PCR assays, to indicate whether the symbiont causes embryonic MK.

### Estimating endosymbiont presence and prevalence

To test for the presence of bacterial endosymbionts in the two *Adalia* species, DNA was first isolated from either whole adult or leg tissue of ladybirds that had been fed on an artificial diet for 48 h prior to being flash frozen in liquid nitrogen. Tissue was homogenized and DNA template was purified from the homogenate following the Promega Wizard Genomic DNA Purification protocol (www.promega.com). PCR amplification of the host mitochondrial DNA CO1 region (primers HCO/LCO [24]) was carried out to confirm successful DNA extraction. Samples were then tested for *Spiroplasma, Rickettsia* and *Wolbachia* infection *via* PCR assays alongside known positive and negative control samples. Primers (1 µl of 20 pmol µl^–1^) amplifying *Spiroplasma* (Haln1/MGSO [25]) *Rickettsia* (Ri_Meg17kD_F/ Ri_Meg17kD_R [26]) and *Wolbachia* (81 F/ 691R [27]) were used in separate reactions with the following reagents: 7.5 µl Promega Hotstart GoTaq, 5.5 µl of RO water and 1 µl DNA template. PCR primers and conditions are available at https://doi.org/10.6084/m9.figshare.21915558 [1]. The PCR products were run on a 1.5 % agarose gel with 3 µl of Midori Green added to allow visualization of amplicons under UV light.

The resulting amplicons were sequenced from a subset of PCR-positive samples to confirm symbiont identity. To this end, PCR products were purified via the Bioline Isolate II Genomic DNA Kit protocol (www.bioline.com). Each sample was washed using CB buffer, CW buffer and then resuspended in water. Purified samples were sent along with the MGSO primer for *Spiroplasma* identification and the HCO primer for CO1 insect DNA, for onward Sanger sequencing by Eurofins (www.eurofins.co.uk). Similar sequences were searched using NCBI blast (https://blast.ncbi.nlm.nih.gov/Blast.cgi), then aligned and compared in Geneious Prime (v2021.2.2, https://www.geneious.com).

### Whole-genome sequencing

Symbiont presence in wild caught specimens can derive from the specimen itself or from cobiont taxa such as parasitic wasps, nematodes, or phoretic mites that are on or in the focal individual (e.g. [28]). Cobionts can be detected from whole-genome-sequencing data, which then additionally allows draft genomes of the symbionts themselves to be constructed. We therefore obtained genomic DNA sequence for the *A. decempunctata* specimen infected with *Spiroplasma *. DNA isolated from the infected male was sent to Novogene (www.novogene.com) for Illumina sequencing. A total of 132.7 million raw reads were produced (150 bp PE, Q20 of 95.4 %). Low-quality reads, reads containing Ns, and short reads were removed from the dataset, and adaptors were trimmed from reads, using fastp and default settings (v0.23.2 [29],) to produce a trimmed and filtered dataset (65.8M forward and reverse reads, with Q20 of 96.8 and 94.9 %, respectively). *Spiroplasma* presence was confirmed in this sample using Phyloflash (v3.4 [30],) on the trimmed reads.

Trimmed reads were *de novo* assembled using megahit (v1.2.9 [31]), to produce a draft metagenomic assembly containing both eukaryotic and *Spiroplasma* sequences. Trimmed reads were also mapped back to the draft metagenomic assembly using BWA-MEM2 (v2.0pre2 [32]) to obtain coverage information. Using taxonomic assignment data from Blobtools2 (v3.0.0 [33]) and local blast +searches (v2.2.29 [34]), the metagenomic assembly was split into separate organism draft genome assemblies. To assess completeness and contamination of the draft genome assemblies, BUSCO (v5.2.2 [35]) scores were calculated, using appropriate databases for the taxonomic group. The raw Illumina sequencing data has been submitted as SRA to NCBI (accession: SRR23019831).

### *Spiroplasma* genome annotation

The draft *Spiroplasma* genome obtained from sequencing the infected *A. decempunctata* individual was annotated using prokka (v1.14.6 [36]) and Interproscan5 (v5.59–91.0 [37]). We also specifically examined the genome for the presence of toxin genes that are considered candidates for the mechanism of MK (i.e. ankyrin repeats and OTU-like cysteine proteases, as found in the plasmid-encoded SPAID protein [38]) or protective phenotypes [i.e. genes encoding ribosomal inactivating proteins (RIPs) originally identified in *S. poulsonii *, which damage the ribosomes of parasitic wasps and nematodes of *Drosophila* [39–41]]. We also searched for high-mobility group (HMG) box domain proteins that have been implicated recently in *Spiroplasma *-induced CI [42]. To this end, we used UniProt alignments downloaded from the Pfam database (pfam-legacy.xfam.org) for protein families RIP (PF00161) and OTU (PF02338). These alignments were then used in hmmer (v3.3.2, hmmer.org), with the prokka CDS as queries. Coding sequences containing ankyrin repeats or HMG box domains were identified in the Interproscan5 annotation output. SignalP-6.0 (https://services.healthtech.dtu.dk/service.php?SignalP-6.0) was utilized to identify signal peptides within the CDS, particularly in the genes of interest.

### *Spiroplasma* phylogeny

A local blast+ search of the draft *Spiroplasma* genome using the Sanger-sequencing short sequence of the *Spiroplasma* 16S rRNA gene as query was used to identify the full sequence of the gene. Nucleotide sequence of 16S rRNA genes from other *Spiroplasma* strains were obtained from (a) *Spiroplasma* 16S deposits in NCBI GenBank, including the full length 16S gene sequence of *Spiroplasma* infecting *Adalia bipunctata* (accession: AJ006775) or (b) whole-genome sequence data of *Spiroplasma* (the 16S sequence was extracted using a local blast +search of the genomes of *Spiroplasma* infecting *Lariophagus distinguendus* (accession: GCA_023846195) and *Nebria riversi* (accession: GCA_018831625). These were then aligned using muscle (v3.8.425 [43]). The relatedness of strains was estimated using a maximum-likelihood-based (ML) method, based on the GTR+F+I+I+R2 model chosen by ModelFinder [44], using IQTree (v 2.2.0.3 COVID-edition [45], with 1000 Bootstrap replicates calculated to assess internal branch support. The 16S rRNA sequence from *Mycoplasma genitalium* was used to root the tree (accession: OM509887).

## Results

### Male-killer prevalence in *Adalia* ladybirds

No *Wolbachia* or *Rickettsia* infected specimens were identified in our samples of either *A. bipunctata* or *A. decempunctata*. In contrast, both *A. bipunctata* and *A. decempunctata* were found to be infected at low prevalence with *Spiroplasma* (18 and 5 % of individuals, respectively; Table 1; individual data is available at https://doi.org/10.6084/m9.figshare.21865035 [2]). *Spiroplasma* in *A. bipunctata* was detected solely in female individuals (4 of 22 females vs 0 of 15 males), and was recovered from template derived from leg material. However, statistical analysis cannot reject the null hypothesis of equal prevalence between sexes (Fisher's exact test, *P*=0.13). In *A. decempunctata*, the single *Spiroplasma *-infected individual was male. Sanger sequencing of the *Spiroplasma* 16S rRNA gene from two *A. bipunctata* and the single *A. decempunctata* sample identified as positive for infection from the PCR assay confirmed the presence of an *ixodetis* group *Spiroplasma* strain in both species. The 335 bp of *Spiroplasma* 16S rRNA sequenced from both *Adalia* species were 100 % identical, and had a 99.7 % sequence similarity to *Spiroplasma ixodetis *, known to infect diverse arthropods and which can cause MK in some species [20, 25, 46, 47]. The three partial *Spiroplasma* 16S rRNA sequences were deposited in GenBank (accessions: OQ271402-OQ271404).

**Table 1.** Symbiont infection status of *A. bipunctata* and *A. decempunctata* partitioned by host sex, with infection status determined through PCR assay

Species	Sex	*N*	Uninfected	* Spiroplasma *	* Wolbachia *	* Rickettsia *
*Adalia bipunctata*	Male	15	15	0	0	0
	Female	22	18	4	0	0
*Adalia decempunctata*	Male	20	19	1	0	0
	Female	20	20	0	0	0

### Spiroplasma does not cause embryonic MK in *A. bipunctata*

Of the 20 female *A. bipunctata* that laid eggs, four were later determined by PCR assay to be infected with *Spiroplasma *. Of these four, none had egg-hatch rates that were indicative of embryonic MK (where the males die as embryos, resulting in *c.* 50 % egg-hatch rate) (Table 2). Comparisons of the egg-hatch rates from *Spiroplasma *-infected females vs uninfected females did not reject the null hypothesis of equal egg-hatch rate in *Spiroplasma* infected *vs* uninfected females (Mann–Whitney *U* test: N1=16, N2=4, *U*=30 *P*=0.89).

**Table 2.** Egg-hatch rates from *A. bipunctata* females partitioned by *Spiroplasma* infection status

Ladybird	* Spiroplasma* infection	Eggs laid	Eggs hatched	HR
AbipLV04	Yes	97	86	0.89
AbipLV17	Yes	18	11	0.61
AbipLV20	Yes	35	34	0.97
AbipGB02	Yes	124	116	0.94
AbipLV02	No	123	122	0.99
AbipLV03	No	88	81	0.92
AbipLV08	No	33	29	0.88
AbipLV09	No	142	115	0.81
AbipLV10	No	30	25	0.83
AbipLV11	No	6	0	0
AbipLV12	No	53	51	0.96
AbipLV16	No	50	41	0.82
AbipLV18	No	13	13	1
AbipLV19	No	14	10	0.71
AbipWL01	No	20	19	0.95
AbipWL02	No	114	98	0.86
AbipWL03	No	116	110	0.95
AbipOX01	No	67	64	0.96
AbipOX02	No	100	86	0.86
AbipGB01	No	49	31	0.63

HR, Egg hatch rate.

### Identification of novel associations in *A. decempunctata*

BUSCO analysis of the initial draft metagenome assembly of the *Spiroplasma* infected *A. decempunctata* sample, using the Insecta database, revealed that a large proportion of the BUSCOs were duplicated (*D*), indicating contamination with DNA from a different insect (BUSCO insecta_odb10: C:98.3 %[S:50.8 %,D:47.5 %],F:1.3 %,M:0.4 %,n:1367). Taxonomic assignment of the assembly contigs revealed the presence of substantial genomic sequence from two further organisms in addition to the ladybird and *Spiroplasma* bacteria: a hymenopteran and a *Beauveria* fungus. To identify the hymenopteran further, a local blast+ search of the assembly using an *A. decempunctata* COI gene sequence (NCBI accession: KU917463) was undertaken and identified the presence of an arthropod non-*Adalia* COI sequence as well as the *A. decempunctata* COI gene. This non-*Adalia* sequence was used as a query against the complete bold coi database (www.boldsystems.org), which identified the closest affiliation with a parasitic wasp of the genus *Centistes* (84.6 % similarity).

Whilst it was not possible to separate the two insect genomes satisfactorily, as neither the ten-spot ladybird or the *Centistes* wasp have sufficient existing genomic resources to enable this, a draft genome of the *Beauveria* genome could be assembled (accession: SAMN32746591). This assembly consists of 3480 contigs totalling 3 353 9846 bp (33.5 Mb), with an N50 of 24 297 and GC content of 52.47 %. The draft genome is 92.5 % complete (BUSCO hypocreales_odb10: C:92.5 %[S:92.1 %,D:0.4 %],F:3.9 %,M:3.6 %,n:4494). A genome size of 33.5 Mb is comparable to published *Beauveria* genomes; strains of the entomopathogenic fungus *Beauveria bassiana* have genomes in the range of 33–39 Mb [48].

### *Spiroplasma* genome assembly and annotation

The draft genome of the *Spiroplasma* infecting *A. decempunctata* consists of 55 contigs, including six contigs that are putative plasmids or partial plasmids (accession: SAMN32746590). Total size is 9 65 424 bp with a GC (%) of 26.77 and N50 of 23 759 bp. Genome completeness is estimated to be 96.7 % according to BUSCO, with three missing BUSCOs (alanine–tRNA ligase, rRNA (cytidine-2′-O-)-methyltransferase and 50S ribosomal protein L7/L12). However, prokka identified all three among the CDS annotated, and so the draft genome is near complete. In all, annotation using prokka revealed there to be 937 CDS (380 annotated), 27 tRNAs, three rRNAs (5S, 16S and 23S) and one tmRNA within the main *Spiroplasma* contigs, and 65 CDS (three annotated) within the six putative plasmid contigs.

No genes carrying RIP toxin domains were identified in the *Spiroplasma* genome, however two CDS contain OTU-like cysteine protease domains. Interestingly, both sequences are closely related to *Spiroplasma* homologues including that recently published from a CI-inducing *Spiroplasma* infecting the parasitic wasp *Lariophagus distinguendus* [42]. Unlike for the *Spiroplasma* MK candidate gene, SPAID, the CDS identified here containing OTU-like cysteine protease domains do not additionally contain ankyrin repeats. However, several other CDS-containing ankyrin repeats were detected; there were ankyrin repeat domains in 11 CDS in the main genome, and a further two among the plasmid CDS. Also of interest is that the *Spiroplasma* genome plus plasmids encode a total of five proteins containing HMG box domains that are very rare in bacteria, though found in several ixodetis group *Spiroplasma *. SignalP analysis reveals that none of the genes of interest contained signal peptides. Annotations are available at https://doi.org/10.6084/m9.figshare.21865113 [3].

### *Spiroplasma* phylogeny

**Fig. 1.**
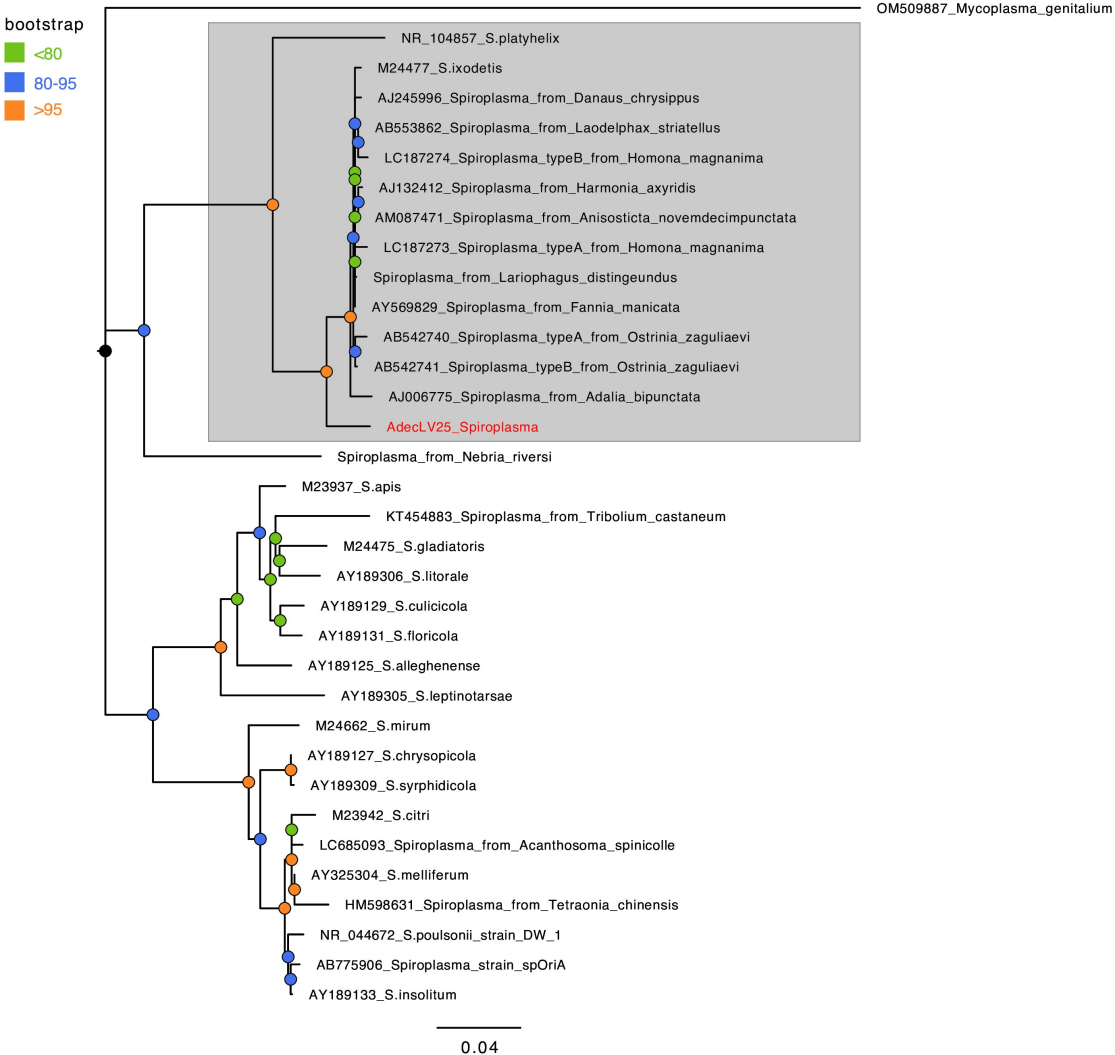
Phylogenetic tree of *Spiroplasma* constructed using full-length 16S rRNA gene sequences as estimated in IQTree. The *Spiroplasma* 16S rRNA sequence obtained from sequencing *Adalia decempunctata* is highlighted in red. Bootstrap support is given by node colour. Scale bar indicates nucleotide divergence along branches. The *ixodetis* clade, to which the *Spiroplasma* from *A. decempunctata* belongs, is indicated by the grey shaded region. The 16S rRNA gene sequence of *Mycoplasma genitalium* was used to root the tree.

Analysis of the complete 16S rRNA gene obtained from Illumina sequencing of the *A. decempunctata* specimen revealed that this strain of *Spiroplasma* resided within the *ixodetis* clade. However, the *A. decempunctata* derived *Spiroplasma* 16S rRNA was distinct to that of the *Spiroplasma* infecting *A. bipunctata* (Fig. 1). The full 16S rRNA sequence of the *A. decempunctata Spiroplasma* was deposited in GenBank (accession: OQ271406).

## Discussion

Spatial variation in heritable symbiont presence is well known in insects [49] such that a full account of symbionts within a species requires analysis of multiple populations. In this paper, we examined the Merseyside population of two ladybirds, *A. bipunctata* (known in other populations studied to be infected with *Wolbachia, Rickettsia* or *Spiroplasma* depending on population sampled, e.g. [20]) and *A. decempunctata* (known to carry MK *Rickettsia* in Germany [21]).

In *A. bipunctata*, a *Spiroplasma ixodetis* relative was found in four female individuals. The *Spiroplasma* was identical in 16S rRNA sequence to the previously reported maternally inherited strain found in *A. bipunctata* [12, 19]. Because of this prior work, it is most parsimonious to presume the strain is an associate of the ladybird rather than a cobiont. However, in contrast to previous laboratory studies, embryonic MK was not observed in infected females from this population, with infected females producing clutches with high egg-hatch rates. This observation, alongside previous PCR screens indicating *Spiroplasma* infection in both male and female *A. bipunctata* from Scotland [50] indicate *Spiroplasma* is not an early male-killer in UK *A. bipunctata* (our data), and that at least some male hosts can survive *Spiroplasma* infection. To determine whether the strain of *Spiroplasma* present is alternatively a late male-killer (as observed in planthoppers [47]), clutches would need to be reared through to maturity. Additionally, transinfecting the *Spiroplasma* onto a Scandinavian genetic background known to support MK would allow us to establish whether phenotype differences are associated with host genetic background (as found for other MK symbiont/host interactions [14, 51]) or symbiont differences (as found in tea tortrix moths [52]). It would also be interesting to determine whether the strain expresses an alternative phenotype such as parasite protection. It is notable that *Rickettsia* infection was not observed in Merseyside *A. bipunctata*, despite being reported in populations from both Southern UK and Scotland [50]. However, *Rickettsia* in *A. bipunctata* commonly exists at low prevalence (5–10 % of females [19]) and may be present in Merseyside but not in our sample of beetles.

Previous work on ten-spot ladybirds revealed male-killing *Rickettsia* in 5–10 % of female beetles collected from Germany [21]. We did not find evidence for *Rickettsia* in Merseyside ten-spot ladybirds, but again the relatively small number of females collected makes it impossible to exclude presence as a rare associate. More surprisingly, *Spiroplasma* bacteria were detected for the first time in *A. decempunctata*, from a single male individual. To investigate whether it is the same strain as the MK *Spiroplasma* carried by *A. bipunctata*, and whether the field caught specimen carried cobionts that might be an alternative source of the infection, genomic sequence was obtained from this individual. Comparison of the full *Spiroplasma* 16S rRNA gene from the *A. decempunctata Spiroplasma* with those previously sequenced indicated that it is not a sister strain to that previously recorded in *A. bipunctata* but is a novel strain in coccinellids. Assembly of the genome of the *Spiroplasma* identified in *A. decempunctata* revealed that it has a genome at the smaller end of the range (965 kbp) relative to other *Spiroplasma* (780 to 2220,220 kbp); more similar in size to *Spiroplasma platyhelix* infecting the dragonfly *Pachydiplax longipennis* [53, 54].

Unexpectedly, the genome data indicated that the *Spiroplasma* infected *A. decempunctata* was also infected with a parasitoid wasp from the Braconidae genus *Centistes*, representing a new natural enemy record for *A. decempunctata*. Ladybirds are host to a number of parasitoids, the best documented of which is another braconid wasp – *Dinocampus coccinellae* [55–57]. While *Centistes* wasps are known to parasitize beetles including ladybirds [58], their interaction with ladybirds remains poorly characterized. The presence of the wasp within the ladybird host raises the question of whether the *Spiroplasma* infects the wasp or the ladybird. *Spiroplasma* have been recorded as symbionts of parasitoid wasps, including the recent discovery of a novel CI-inducing *Spiroplasma* strain in the wasp *Lariophagus distinguendus* [42]. Future work would establish the host for the *Spiroplasma* through a broader collection of wasps and ladybirds, and fluorescence *in situ* hybridization (FISH) analysis to establish the site of symbiont infection.

The phenotype of the *Spiroplasma* identified in the *A. decempunctata* metagenome remains unknown. Should it be a symbiont of the ladybird, its presence in a male suggests either incomplete MK or a non-MK *Spiroplasma* strain. Annotation of the *Spiroplasma* genome reveals that it encodes two OTU-like cysteine proteases closely related to a homologue found in the CI-inducing *Spiroplasma* of the parasitic wasp *Lariophagus distinguendus* [42]. Similarly, the genome also contains multiple high-mobility group (HMG) box proteins as does the *Spiroplasma* in *Lariophagus distinguendus.* HMG box domain containing genes are common in eukaryotes, but very rare in bacteria. Indeed, all records to date are from *ixodetis* group *Spiroplasma *. In addition, 13 CDS were identified to contain ankyrin-repeat domains, which commonly mediate interactions with host eukaryotic proteins and are important components of establishing symbiosis and symbiont phenotype [59]. This genome therefore provides several candidates for further investigation.

This case study highlights a general issue of the accuracy of screening programmes where field collected material is analysed solely on the basis of PCR assays – namely that an individual of a species may return PCR positive, but this is a false positive for the specimen and/or species because it arises from a cobiont. Our study complements others, with recent cases identifying *Arsenophonus* and *Wolbachia* amplifying from cobiont material rather than the identified collected arthropod species [28, 60]. The problem of false-positive reports from cobionts is likely greatest for infections that are apparently rare within a species, because cobionts will tend to be in a fraction of individuals rather than all individuals. Whilst rare symbiont infections certainly exist in insects, PCR screening data alone should therefore not be used to establish their presence. Rather, low prevalence infections require corroboration either from whole specimen genome sequence (excluding a cobiont from being in the material), laboratory breeding work using individuals uninfected with the cobiont, or additional FISH studies (localizing symbiont to host).

Genome sequencing of the *A. decempunctata* individual also revealed the presence of a parasitic fungus from the genus *Beauveria*. These fungi are facultative pathogens commonly known to infect ladybirds via exposure in leaf matter and soil during overwintering [61, 62]. *Beauvaria* are known to be able to infect and cause mortality in *Adalia* in laboratory exposure trials [63]. However, despite being documented as a major cause of mortality in *Coccinellidae* [64], mycosed *Adalia* have not been reported in the field, leading to the widespread belief that their above ground overwintering site makes exposure unlikely. Our infected individual was an individual collected from an above ground overwintering site (gravestone) and indicates *Beauvaria* does infect this species in the field despite being spatially separate from leaf litter sources of infection.

In conclusion, we screened for the presence of three common male-killers in two *Adalia* ladybirds. While our data extends previous work in *A. bipunctata* in describing *Spiroplasma* presence in a new population, we also show that this strain does not cause embryonic male-killing, contrasting to its phenotype in *A. bipunctata* from Continental European and Scandinavian populations. We also present the discovery of a novel strain of *Spiroplasma* in *A. decempunctata*, quite unlike those previously sequenced from ladybird beetles. Genomic analysis of the metagenome of *A. decempunctata* unexpectedly revealed the presence of two further organisms infecting the ladybird: a *Centistes* parasitoid wasp, and the entomopathogenic fungus *Beauveria*. These cobiont records highlight the complexity of ascertaining the infection status of field-collected individuals. Moreover, it emphasizes that organisms rarely, if ever, develop, live, or evolve without direct interaction with a multitude of others.
